# Intracoronary nicorandil improves coronary microcirculatory function after primary PCI in first-episode STEMI: an angiography-derived evaluation using AMR and QFR

**DOI:** 10.3389/fcvm.2026.1786130

**Published:** 2026-04-22

**Authors:** Qing Yu, Jie Lin, Jiashi Ding, Feifei Ye, Peng Li, Shikun Chen, Yiling Zhan, Wenqin Cai, Jiaqi Ou

**Affiliations:** 1 Department of Cardiology, Shaowu Municipal Hospital of Fujian Province, Shaowu, Fujian, China; 2 The First Affiliated Hospital of Fujian Medical University, Fuzhou, Fujian, China

**Keywords:** angiographic microcirculatory resistance, coronary microcirculation, nicorandil, primary percutaneous coronary intervention, quantitative flow ratio, ST-segment elevation myocardial infarction

## Abstract

**Background:**

Microvascular dysfunction remains a major driver of adverse outcomes after ST-segment elevation myocardial infarction (STEMI) despite successful restoration of epicardial patency by primary percutaneous coronary intervention (PCI). Nicorandil has nitrate-like vasodilatory properties and ATP-dependent potassium channel (KATP)-opening properties, effects that may improve reperfusion physiology. However, its effects on wire-free, angiography-derived measures of microvascular function are not well defined. We evaluated whether intracoronary nicorandil administered during primary PCI improves angiography-derived microvascular function assessed by angiographic microcirculatory resistance (AMR) and quantitative flow ratio (QFR).

**Methods:**

In this prospective, single-center randomized trial, 63 patients with first-episode STEMI undergoing primary PCI were allocated 1:1 to intracoronary nicorandil (2 mg after guidewire crossing; *n* = 32) or control (standard PCI; *n* = 31). The prespecified primary endpoint was final post-PCI AMR. QFR-derived indices, reperfusion measures [Thrombolysis in Myocardial Infarction (TIMI) flow grade, no-reflow, ST-segment resolution], hemodynamics, biomarkers, and clinical events were analyzed as secondary/exploratory outcomes, with multiplicity controlled using the Benjamini–Hochberg false discovery rate (FDR) where applicable.

**Results:**

Baseline characteristics were balanced between groups. Compared with control, intracoronary nicorandil was associated with a higher rate of post-PCI TIMI grade 3 flow (96.9% vs. 74.2%; overall *P* = 0.013). Final AMR was significantly lower in the nicorandil group (1.4 ± 0.5 vs. 2.7 ± 0.5; *P* < 0.001). Vessel QFR and change in QFR (*Δ*QFR) also favored nicorandil (both *P* < 0.001), whereas residual QFR showed only a borderline/non-significant difference after FDR correction (raw *P* = 0.027; q = 0.051). Peak creatine kinase-MB (CK-MB) and cardiac troponin I (cTnI) were numerically lower with nicorandil but not statistically different; left ventricular ejection fraction (LVEF) at 1 week was similar between groups. Rates of intra-procedural hypotension, in-hospital major adverse cardiovascular events (MACE), and MACE at 3-month follow-up did not differ.

**Conclusions:**

In this pilot randomized study, intracoronary nicorandil administered during primary PCI was associated with improved angiography-derived surrogate indices of microvascular resistance and epicardial physiology without an observed increase in peri-procedural hemodynamic instability or short-term adverse events. These findings should be interpreted as exploratory and warrant confirmation in larger multicenter studies with longer follow-up and outcome-linked validation.

## Introduction

ST-segment elevation myocardial infarction (STEMI) is a severe presentation of acute myocardial infarction, and cardiovascular disease remains a leading cause of morbidity and mortality worldwide. Current guidelines recommend primary percutaneous coronary intervention (PCI) as the preferred reperfusion strategy for eligible patients with STEMI (1, 2). Although primary PCI restores epicardial coronary patency in most patients, optimal clinical outcomes are not consistently achieved. A considerable proportion of patients exhibit persistent microvascular dysfunction despite achieving Thrombolysis in Myocardial Infarction (TIMI) grade 3 flow, a condition known as no-reflow. This inadequate microcirculatory reperfusion is strongly associated with ongoing myocardial ischemia, larger infarct size, adverse left ventricular remodeling, and worse short- and long-term clinical outcomes (3). Consequently, improving coronary microvascular function beyond epicardial recanalization remains a critical unmet need in contemporary STEMI care.

Nicorandil, a hybrid vasodilator with nitrate-like effects and ATP-dependent potassium channel (KATP) –opening properties, is widely used as an adjunctive therapy in acute myocardial infarction. Nicorandil induces dilation of both epicardial coronary arteries and resistance microvessels, thereby enhancing myocardial perfusion, attenuating ischemia-reperfusion injury, and potentially reducing myocardial necrosis. Prior studies have suggested that intracoronary nicorandil administered during primary PCI may improve reperfusion; however, these studies primarily assessed surrogate markers such as TIMI flow grade, myocardial blush grade, or enzymatic infarct size. Whether intracoronary nicorandil can directly and quantitatively reduce coronary microvascular resistance during primary PCI for STEMI remains inadequately defined (3, 4).

Recent advances in angiography-based physiological assessment enable evaluation of coronary function without pressure wires or pharmacological hyperemia. Quantitative flow ratio (QFR) provides a wire-free estimate of epicardial coronary physiology and has been validated against pressure-wire-derived fractional flow reserve (FFR) (5, 6). Angiography-derived indices of microvascular resistance have been evaluated against invasive index of microcirculatory resistance (IMR), and more recent STEMI-specific studies further support the feasibility and prognostic relevance of angiographic microcirculatory resistance (AMR) for assessing post-PCI microvascular dysfunction (7–10). The combined application of QFR and AMR allows integrated assessment of both epicardial and microvascular components of coronary reperfusion in routine clinical practice.

Accordingly, this prospective randomized study investigated whether intracoronary nicorandil administered during primary PCI improves coronary microvascular function, assessed by AMR, and epicardial physiological recovery, assessed by QFR, in patients with first-episode STEMI. We also evaluated procedural safety and short-term clinical outcomes to further define the therapeutic role of nicorandil in optimizing reperfusion quality.

## Methods

### Study design and population

This prospective, single-center, randomized controlled trial consecutively enrolled patients presenting with first-episode STEMI at Shaowu Municipal Hospital between December 1, 2023, and October 1, 2025. All enrolled patients underwent emergent primary PCI. We included only patients with angiographic images of sufficient quality obtained before and after PCI to permit reliable QFR and AMR analyses.

### Inclusion criteria

- Age ≥18 years
- First-time diagnosis of STEMI according to current clinical guidelines
- Symptom onset within the recommended therapeutic time window for primary PCI
- Clearly identifiable infarct-related artery (IRA) suitable for urgent revascularization
- Adequate angiographic image quality to allow QFR and AMR computation

### Exclusion criteria

- Prior myocardial infarction or a history of coronary artery bypass grafting
- Significant valvular heart disease or nonischemic cardiomyopathy
- Clinically severe hepatic or renal dysfunction, assessed on the basis of liver/renal function testing and relevant medical history at screening. Renal function assessment included estimated glomerular filtration rate (eGFR).
- Active malignancy or systemic infection
- Cardiogenic shock requiring mechanical circulatory support
- Incomplete angiographic data or suboptimal image quality precluding QFR or AMR analysis
- Inability or refusal to provide written informed consent

### Randomization and consent

Eligible patients were randomized in a 1:1 ratio to the nicorandil group or the control group using an Excel-generated random number sequence prepared by designated study personnel. Allocation concealment was ensured using sealed opaque envelopes, which were opened after successful guidewire crossing of the culprit lesion and at the time of treatment assignment.The Ethics Committee of Shaowu Municipal Hospital approved the study protocol (Approval No. 2023-7), and written informed consent was obtained within the Chest Pain Center workflow as early as feasible after initial stabilization and prior to randomization and study-specific administration. When patients lacked decision-making capacity in the emergency setting, written informed consent was obtained from a legal guardian/next of kin in accordance with institutional policy and Ethics Committee approval. Patients for whom valid consent (patient or surrogate) could not be obtained within the required timeframe were not enrolled.

### PCI procedure and nicorandil administration

All patients received guideline-directed medical therapy, including dual antiplatelet therapy, anticoagulation, and standard secondary prevention medications. Operators performed PCI via radial or femoral arterial access according to clinical judgment and anatomical considerations.

Infarct-related arteries were identified based on electrocardiographic findings, angiographic features, and overall clinical assessment. Balloon predilation, stent implantation, postdilation, and aspiration thrombectomy were performed when clinically indicated.

### Intervention and control treatment

#### Nicorandil group

Immediately after successful guidewire passage across the culprit lesion, operators administered 2 mg of nicorandil diluted in saline intracoronarily into the infarct-related artery through the guiding catheter or a microcatheter. Operators then completed PCI according to standard interventional practice.

#### Control group

Patients assigned to the control group underwent conventional primary PCI without intracoronary administration of nicorandil.

#### Adjunctive therapies

For all patients undergoing primary PCI, the use of adjunctive therapies, including glycoprotein IIb/IIIa inhibitors, thrombus aspiration, or additional vasodilators, was permitted at the operator’s discretion, regardless of group assignment.

#### Angiographic assessment and derivation of QFR and AMR

Operators performed coronary angiography before and after PCI using optimized projection angles and standardized frame acquisition rates to ensure adequate image quality for three-dimensional vessel reconstruction and subsequent analysis.

#### Epicardial flow assessment

Epicardial flow was assessed offline after the index procedure using stored angiographic recordings. Two experienced interventional cardiologists, blinded to treatment allocation, independently evaluated:

- Thrombolysis In Myocardial Infarction (TIMI) flow grade
- The presence of no-reflow, defined as persistent TIMI flow ≤ 2 or impaired myocardial blush in the absence of mechanical obstruction.

Disagreements between the two reviewers were resolved by consensus.

#### ST-segment resolution

ST-segment resolution (STR) was assessed using 12-lead electrocardiograms obtained 60 min after primary PCI. Two cardiologists, blinded to treatment allocation, independently classified STR as complete (>70%), partial (30%–70%), or absent (<30%). Disagreements were resolved by consensus.

#### QFR and AMR analysis

In the acute STEMI setting, routine invasive physiology (FFR/IMR) may be constrained by procedural time, hemodynamic instability, and the need for pressure wires and/or hyperemic agents. Accordingly, we used angiography-derived physiological indices as a practical wire-free approach based on standard angiographic acquisitions, enabling immediate post-PCI assessment within real-world emergency workflows. Post-PCI angiographic images were analyzed offline using the AngioPlus® system (BoDong Medical Technology, Shanghai, China; software version 2.1.1.0). Analyses were performed on de-identified DICOM angiograms using standardized reconstruction settings according to the manufacturer's workflow.

- We derived Vessel QFR from three-dimensional vessel reconstruction and contrast frame count–based flow velocity estimation.
- We calculated AMR as an angiography-based surrogate of microvascular resistance using QFR and physiological flow parameters.
- Additional physiological indices included *Δ*QFR, representing the proximal-to-distal pressure gradient, and residual QFR, indicating post-PCI epicardial physiological significance.
- AMR is derived from angiography-based flow modeling and therefore is not independent of QFR; accordingly, we interpret concordant changes in AMR and QFR as internally consistent angiography-derived physiological signals rather than fully independent measures.
- Given the acute STEMI setting, angiography-derived indices provide a practical wire-free approach when routine invasive IMR/FFR acquisition may be constrained by time, hemodynamics, or procedural complexity.

Previous validation studies have shown that QFR has good diagnostic performance compared with pressure-wire–derived FFR, whereas angiography-derived indices of microvascular resistance have been evaluated against invasive IMR. In addition, more recent STEMI-specific studies support the feasibility and prognostic relevance of AMR for the assessment of post-PCI microvascular dysfunction (5–10). To reduce variability, angiographic acquisition was standardized with respect to frame rate, projection quality, and contrast injection, and only cases meeting prespecified image-quality criteria were included in the QFR/AMR analysis. All QFR/AMR measurements were performed offline after the index procedure by trained analysts who were blinded to treatment allocation. A subset of cases was reanalyzed to assess reproducibility, and discrepant results were resolved according to a prespecified adjudication workflow. Although analyses were not performed in a central core laboratory, these quality-control procedures were implemented to minimize measurement variability.

#### Laboratory testing and echocardiographic assessment

Baseline laboratory testing included lipid profile, fasting glucose, glycated hemoglobin, renal function indices, N-terminal pro–B-type natriuretic peptide (NT-proBNP), creatine kinase-MB (CK-MB), cardiac troponin I (cTnI), and D-dimer. Laboratory analyses were performed in the hospital central laboratory according to standard laboratory procedures. NT-proBNP was measured using a magnetic microparticle chemiluminescence assay (Autobio), whereas cTnI was measured using a chemiluminescent immunoassay (Access hsTnI). Renal function was assessed using routine laboratory measurements, including serum creatinine and estimated glomerular filtration rate (eGFR).

Transthoracic echocardiography was performed using a Philips E33 ultrasound system equipped with a cardiac transducer. Chamber quantification and left ventricular systolic function assessment were performed according to standard echocardiographic recommendations, and left ventricular ejection fraction (LVEF) was measured using the biplane Simpson method, where feasible.

#### Clinical endpoints and follow-up

Primary Functional Endpoints

- Final post-PCI AMR

Secondary Endpoints

- Post-PCI Vessel QFR
- *Δ*QFR and residual QFR
- ST-segment resolution categories
- No-reflow/TIMI flow grade
- Intraoperative hypotension
- In-hospital major adverse cardiovascular events(MACE) and 3-month MACE

#### Definitions

Intraoperative hypotension: systolic blood pressure < 90 mmHg for ≥ 30 s or requiring vasopressor support.

MACE: heart failure, malignant ventricular arrhythmias, recurrent myocardial infarction, target-vessel revascularization, or all-cause mortality.

We conducted clinical follow-up at 3 months by outpatient visit or structured telephone interview performed by cardiologists blinded to treatment allocation.

#### Sample size estimation

*A priori* sample size estimation was performed for the prespecified primary endpoint (final post-PCI AMR) using a two-sample comparison of means with a 1:1 allocation ratio. Based on prior reports on AMR variability, the standard deviation (SD) was assumed to be 0.5 mmHg·s/cm (11). A conservative clinically meaningful between-group difference of *Δ* = 0.4 mmHg·s/cm was prespecified, informed by prior STEMI primary PCI data showing clinically relevant AMR separation between risk strata (12). With a two-sided *α* of 0.05% and 80% power, 25 participants per group (50 total) were required. Assuming a 10% dropout rate, the target sample size was approximately 56 participants. The final enrolled sample (*n* = 63) exceeded this estimate.

#### Statistical analysis

All analyses were performed in R version 4.4.2 (2024-10-31) in RStudio 2024.12.0. Continuous variables were assessed for normality using the Shapiro–Wilk test and are presented as mean ± SD or median (interquartile range, IQR), as appropriate. Between-group comparisons were performed using Student's t-test for approximately normally distributed variables and the Wilcoxon rank-sum test for non-normally distributed variables. Categorical variables are presented as counts (percentages) and were compared using the *χ*^2^ test or Fisher's exact test, as appropriate.

The prespecified primary endpoint was final post-PCI AMR, tested at a two-sided significance level of 0.05. All other physiological, angiographic, biomarker, and clinical outcomes were considered secondary/exploratory.

To address multiplicity across secondary/exploratory endpoints, we controlled the false discovery rate using the Benjamini–Hochberg procedure, reporting both raw *P* values and adjusted q values where applicable. BH-FDR correction was applied across all prespecified secondary/exploratory endpoints reported in Tables 1, 2 (physiological indices, hemodynamics, and clinical events). For secondary/exploratory outcomes, statistical inference was primarily based on q values (q < 0.05), while effect estimates with 95% confidence intervals are provided to aid interpretation.

**Table 1:** Procedural characteristics and myocardial reperfusion outcomes.

Variable	Control	Nicorandil	*P*-value	*q*-value
Pre-PCI TIMI flow grade, *n* (%)			**0.273**	**0.491**
Pre-PCI TIMI flow grade: 0	22 (71.0%)	18 (56.2%)	-	-
Pre-PCI TIMI flow grade: 1	5 (16.1%)	3 (9.4%)	-	-
Pre-PCI TIMI flow grade: 2	3 (9.7%)	8 (25.0%)	-	-
Pre-PCI TIMI flow grade: 3	1 (3.2%)	3 (9.4%)	-	-
Post-PCI TIMI flow grade, *n* (%)			**0.013**	**0.034**
Post-PCI TIMI flow grade, *n* (%): 0	0 (0.0%)	0 (0.0%)	-	
Post-PCI TIMI flow grade, *n* (%): 1	0 (0.0%)	0 (0.0%)	-	
Post-PCI TIMI flow grade, *n* (%): 2	8 (25.8%)	1 (3.1%)	-	
Post-PCI TIMI flow grade, *n* (%): 3	23 (74.2%)	31 (96.9%)	-	
ST-segment resolution (*n*, %)			**0.355**	**0.567**
ST-segment resolution: CR	21 (67.7%)	27 (84.4%)	-	
ST-segment resolution: PR	8 (25.8%)	4 (12.5%)	-	
ST-segment resolution: NR	2 (6.5%)	1 (3.1%)	-	
No-reflow phenomenon (n, %)			**0.613**	**0.817**
No-reflow phenomenon: No	29 (93.5%)	31 (96.9%)	-	
No-reflow phenomenon: Yes	2 (6.5%)	1 (3.1%)	-	

Control group (*n* = 31) and nicorandil group (*n* = 32). Values are presented as *n* (%). For multi-category variables, including pre-PCI Thrombolysis In Myocardial Infarction (TIMI) flow grade, post-PCI TIMI flow grade, and ST-segment resolution categories, *P* values represent a single global (2 × k) comparison using the *χ*^2^ test or Fisher's exact test, as appropriate. q values were obtained using the Benjamini–Hochberg false discovery rate correction across prespecified secondary/exploratory endpoints. TIMI, thrombolysis in myocardial infarction; PCI, percutaneous coronary intervention; CR, complete resolution; PR, partial resolution; NR, no resolution. Bold values highlight the key indicators of interest in this study.

**Table 2:** Angiography-derived physiological parameters, hemodynamics, and clinical events.

Variable	Control	Nicorandil	*P*-value	*q*-value
Angiographic and hemodynamic parameters				
Final AMR	2.7 ± 0.5	1.4 ± 0.5	<0.001	-
Vessel QFR	0.6 ± 0.3	0.9 ± 0.1	<0.001	<0.001
*Δ*QFR	0.3 ± 0.3	0.1 ± 0.1	<0.001	<0.001
Residual QFR	0.9 ± 0.1	1.0 ± 0.1	0.020	0.051
Heart rate pre-PCI (bpm)	80.6 ± 15.3	87.5 ± 18.5	0.112	0.358
Heart rate post-PCI (bpm)	74.5 ± 14.8	75.1 ± 10.7	0.559	0.794
BP pre-PCI (mmHg)				
SBP pre-PCI (mmHg)	124.9 ± 24.8	126.1 ± 25.4	0.847	0.895
DBP pre-PCI (mmHg)	102.8 ± 46.6	79.2 ± 14.4	0.380	0.794
BP post-PCI (mmHg)				
SBP post-PCI (mmHg)	115.2 ± 11.9	114.1 ± 18.6	0.780	0.794
DBP post-PCI (mmHg)	70.8 ± 10.5	71.1 ± 8.5	0.895	0.895
Intra-procedural safety, *n* (%)				
Intra-procedural hypotension	9 (29.0%)	6 (18.8%)	0.387	0.709
In-hospital MACE, *n* (%)			**0.695**	**0.695**
Heart failure	8 (25.8%)	9 (28.1%)	1.000	
Malignant ventricular arrhythmia	3 (9.7%)	0 (0.0%)	0.113	
Recurrent myocardial infarction	0 (0.0%)	0 (0.0%)	1.000	
Target vessel revascularization	0 (0.0%)	0 (0.0%)	1.000	
Death	1 (3.2%)	0 (0.0%)	0.492	
MACE at 3 months, *n* (%)			**0.689**	**0.695**
Heart failure	10 (32.3%)	9 (28.1%)	0.788	
Malignant ventricular arrhythmia	0 (0.0%)	0 (0.0%)	1.000	
Recurrent myocardial infarction	1 (3.2%)	0 (0.0%)	0.492	
Target vessel revascularization	0 (0.0%)	0 (0.0%)	1.000	
Death	1 (3.2%)	1 (3.1%)	1.000	

Control group (*n* = 31) and nicorandil group (*n* = 32). Values are presented as mean ± standard deviation (SD) or *n* (%), as appropriate. *P* values were calculated using Student's t-test or the Wilcoxon rank-sum test for continuous variables, and the *χ*^2^ test or Fisher's exact test for categorical variables, as appropriate. q values were obtained using the Benjamini–Hochberg false discovery rate correction across prespecified secondary/exploratory endpoints. The primary endpoint, final angiographic microcirculatory resistance (AMR), was not subjected to multiplicity adjustment. AMR, angiographic microcirculatory resistance; QFR, quantitative flow ratio; BP, blood pressure; SBP, systolic blood pressure; DBP, diastolic blood pressure; bpm, beats per minute; PCI, percutaneous coronary intervention; MACE, major adverse cardiovascular events; SD, standard deviation. Bold values highlight the key indicators of interest in this study.

As exploratory analyses, inadequate ST-segment resolution (≤70%) was modeled using Firth penalized logistic regression due to sparse cells/separation in TIMI categories under maximum-likelihood estimation. Odds ratios (ORs) with 95% confidence intervals are reported. The primary exploratory model included treatment group and age. An extended exploratory model additionally included final post-PCI TIMI flow grade (3 vs. <3). A sensitivity exploratory model further adjusted for baseline pre-PCI TIMI flow (0 vs. ≥1). A further extended exploratory model additionally incorporated post-PCI angiographic microcirculatory resistance(AMR). Because final TIMI flow and post-PCI AMR are post-procedural variables that may lie on the treatment-to-reperfusion pathway, these extended models were considered supplementary exploratory analyses and interpreted cautiously.

Baseline balance between groups was assessed using standardized mean differences (SMD), with SMD < 0.10 indicating acceptable balance.

## Results

### Baseline characteristics

A total of 63 patients with first-episode STEMI undergoing primary PCI were included in the final analysis, with 31 patients assigned to the control group and 32 to the nicorandil group.

Baseline demographic characteristics, cardiovascular risk factors, laboratory parameters, renal function indices, ischemic time variables, and infarct-related artery distribution were broadly comparable between groups, with no statistically significant between-group differences (Table 3).

**Table 3:** Baseline characteristics of patients with ST-segment elevation myocardial infarction undergoing primary PCI.

Variable	Overall (*n* = 63)^1^	Control (*n* = 31)^1^	Nicorandil (*n* = 32)^1^	*P*-value^2^
Demographic characteristics				
Age, years	64 ± 14	65 ± 13	63 ± 14	0.874
Male sex, *n* (%)				0.237
Male	48 (76%)	26 (84%)	22 (69%)	
Female	15 (24%)	5 (16%)	10 (31%)	
Cardiovascular risk factors				
Hypertension, *n* (%)	24 (39%)	13 (43%)	11 (34%)	0.603
Diabetes mellitus, *n* (%)	18 (29%)	6 (19%)	12 (38%)	0.164
Current smoking, *n* (%)	17 (27%)	8 (26%)	9 (29%)	> 0.999
Dyslipidemia, *n* (%)	30 (48%)	16 (52%)	14 (44%)	0.617
Baseline laboratory parameters				
Total cholesterol, mM	4.42 ± 1.01	4.29 ± 0.99	4.55 ± 1.03	0.496
Triglycerides, mM	1.49 [0.97, 2.01]	1.49 [0.86, 2.32]	1.49 [1.09, 1.96]	0.645
LDL cholesterol, mM	2.51 ± 0.78	2.38 ± 0.80	2.63 ± 0.76	0.280
HDL cholesterol, mM	1.12 ± 0.30	1.13 ± 0.27	1.10 ± 0.33	0.523
Fasting glucose, mM	7.00 [5.62, 9.05]	6.94 [5.33, 9.05]	7.06 [5.73, 9.32]	0.573
HbA1c, %	6.00 [5.50, 7.10]	6.00 [5.50, 6.60]	6.00 [5.55, 7.10]	0.690
Renal function				
Urinary microalbumin, mg/L	34 [13, 75]	39 [15, 79]	33 [11, 69]	0.483
Estimated GFR, mL/min/1.73 m^2^	78 ± 26	75 ± 28	82 ± 24	0.299
Serum creatinine, μM	94 ± 50	103 ± 59	86 ± 37	0.132
Ischemic time variables Symptom-to-balloon time, h	5 [4, 10]	5 [3, 11]	5 [4, 10]	0.606
Door-to-balloon time, h	1.40 [1.00, 2.00]	1.40 [1.00, 2.00]	1.40 [1.00, 2.25]	0.798
IRA				0.941
LAD	33 (52%)	17 (55%)	16 (50%)	
LCX	10 (16%)	5 (16%)	5 (16%)	
RCA	20 (32%)	9 (29%)	11 (34%)	

^1^Overall (*n* = 63), control group (*n* = 31), and nicorandil group (*n* = 32). Values are presented as mean ± standard deviation (SD), median [Q1, Q3], or *n* (%), as appropriate. ^2^*P* values were calculated using the Wilcoxon rank-sum test or Fisher's exact test, as appropriate. HbA1c, glycated hemoglobin; GFR, glomerular filtration rate; IRA, infarct-related arter; LAD, left anterior descending artery; LCX, left circumflex artery; RCA, right coronary artery; SD, standard deviation; Q1, first quartile; Q3, third quartile.

Standardized mean differences (SMDs) for all baseline variables were <0.10, supporting adequate baseline balance between groups.

### Procedural characteristics and reperfusion outcomes

Before PCI, the distribution of preprocedural TIMI flow grades (0–3) was comparable between the nicorandil and control groups.

After PCI, epicardial reperfusion differed between groups. The postprocedural TIMI grade distribution showed a significant between-group difference, driven by a higher proportion of TIMI 3 flow in the nicorandil group and a lower proportion of TIMI 2 flow.

The no-reflow phenomenon was infrequent and did not differ significantly between groups. Postprocedural ST-segment resolution (STR) categories did not differ significantly between groups. Complete STR was numerically more common in the nicorandil group, whereas partial and absent STR were less frequent; however, these differences did not reach statistical significance on the global test.

Exploratory Firth penalized logistic regression analyses for inadequate STR (≤70%) are presented in Supplementary Figures S1–S4, Table S1. In the primary exploratory model including treatment group and age, the association between intracoronary nicorandil and inadequate STR was estimated with wide confidence intervals. This relationship was further explored in supplementary models incorporating final post-PCI TIMI flow grade, baseline pre-PCI TIMI flow, and post-PCI angiographic microcirculatory resistance (AMR). Given the limited sample size and the exploratory nature of these analyses, these findings should be interpreted cautiously.

Taken together, these procedural and reperfusion findings should be interpreted as exploratory reperfusion surrogates; confirmation in larger, multicenter studies with longer follow-up and outcome-linked endpoints is warranted.

### Periprocedural myocardial injury and early left ventricular function

Before PCI, baseline markers of myocardial injury and cardiac function, including NT-proBNP, CK-MB, cTnI, and D-dimer, did not differ significantly between the nicorandil and control groups.After PCI, peak myocardial biomarker levels measured during hospitalization showed a consistent numerical reduction in patients receiving intracoronary nicorandil, although these differences did not reach statistical significance.

Peak CK-MB and cTnI levels were numerically lower in the nicorandil group, whereas NT-proBNP measured approximately 1 week after PCI and left ventricular ejection fraction (LVEF) were similar between groups (Table 4).

**Table 4:** Periprocedural myocardial injury markers and early cardiac functions.

Variable	Control (*n* = 31)^1^	Nicorandil (*n* = 32)^1^	*P*-value^2^
NT-proBNP before PCI (pg/mL)	259 [53, 1,869]	90 [11, 1,557]	0.106
NT-proBNP 1 week after PCI (pg/mL)	640 [259, 2,158]	799 [243, 1,574]	0.746
CK-MB pre-PCI (U/L)	38 ± 63	26 ± 46	0.384
CK-MB peak level (U/L)	61 [17, 168]	31 [6, 91]	0.163
cTnI pre-PCI (ng/mL)	3.7 ± 6.9	3.8 ± 9.3	0.959
cTnI peak level (ng/mL)	46 [26, 97]	31 [6, 72]	0.082
D-dimer (g/L)	0.39 ± 0.38	0.55 ± 0.62	0.232
LVEF 1 week post-PCI (%)	52.3 ± 7.1	54.5 ± 6.7	0.209

^1^Control group (*n* = 31) and nicorandil group (*n* = 32). Values are presented as median [Q1, Q3] or mean ± standard deviation (SD), as appropriate. ^2^*P* values were calculated using the Wilcoxon rank-sum test or Welch's two-sample t-test, as appropriate. NT-proBNP, N-terminal pro–B-type natriuretic peptide; PCI, percutaneous coronary intervention; CK-MB, creatine kinase-MB; cTnI, cardiac troponin I; LVEF, left ventricular ejection fraction; SD, standard deviation; Q1, first quartile; Q3, third quartile.

Overall, intracoronary nicorandil was associated with directionally lower myocardial enzyme release and higher early LVEF; however, no statistically significant differences were observed in biomarkers or LVEF during the early post-PCI period.

### Angiography-derived microcirculatory and hemodynamic indices

Angiography-derived physiological analysis showed significant improvement in coronary microcirculatory function in patients treated with intracoronary nicorandil.

Final angiographic microcirculatory resistance (AMR) was lower in the nicorandil group, consistent with reduced angiography-derived microvascular resistance. Quantitative flow ratio (QFR)-derived indices also favored nicorandil, whereas the difference in residual QFR was attenuated after false discovery rate adjustment (Table 2). These findings reflect changes in angiography-derived surrogate indices and should not be interpreted as definitive proof of improved microcirculatory function.

### Short-term safety outcomes and 3-month clinical events

Periprocedural hemodynamic variables, including heart rate and blood pressure before and after PCI, were comparable between groups, with no significant between-group differences after adjustment for multiplicity.

The incidence of intra-procedural hypotension was numerically lower in the nicorandil group, although this difference was not statistically significant. All hypotensive episodes were transient and resolved with conservative management, without the need for urgent intervention or mechanical circulatory support.

Clinical outcomes were also similar between groups. In-hospital MACE did not differ significantly between groups, and MACE at 3-month follow-up remained infrequent and comparable. Overall, in this single-center pilot study, intracoronary nicorandil was not associated with an increased risk of peri-procedural hypotension or short-term adverse cardiovascular events, indicating no safety signal within the limitations of the sample size and follow-up duration. Larger studies with longer follow-up are warranted to more definitively characterize safety and clinical outcomes.

## Discussion

In this prospective randomized study, intracoronary nicorandil administered during primary PCI was associated with improved angiographic reperfusion and angiography-derived physiological surrogate indices of epicardial and microvascular function in patients with first-episode STEMI. This study addresses an important issue in contemporary STEMI care: whether adjunctive pharmacological therapy can quantitatively improve reperfusion physiology beyond angiographic TIMI grade alone. Nicorandil treatment was associated with a higher rate of postprocedural TIMI grade 3 flow, lower final AMR, and improved QFR-based indices. We also observed numerically lower myocardial enzyme release without evidence of increased peri-procedural hypotension or short-term adverse events. Given the modest sample size, single-center design, and short follow-up, these findings should be interpreted as exploratory and hypothesis-generating, and outcome-linked validation in larger studies is warranted.

### Nicorandil enhances quality of epicardial reperfusion during primary PCI

Although primary PCI restores epicardial coronary patency in most patients with STEMI, growing evidence indicates that reperfusion quality - rather than vessel reopening alone - critically determines myocardial salvage and clinical outcomes. In this study, intracoronary nicorandil significantly increased the proportion of patients achieving postprocedural TIMI grade 3 flow. This observation aligns with previous reports showing that nicorandil administration during reperfusion improves coronary perfusion and attenuates microvascular dysfunction (4, 13).

From a mechanistic perspective, nicorandil exerts nitrate-like vasodilatory effects that reduce coronary vasospasm, while activation of KATP channels induces relaxation of vascular smooth muscle in both epicardial arteries and resistance microvessels. By simultaneously lowering vascular tone and enhancing downstream microvascular conductance, nicorandil likely improves the efficiency and completeness of epicardial reperfusion observed after PCI.

### Nicorandil improves coronary microvascular function as assessed by AMR and QFR

A key strength of this study lies in the integrated use of angiography-derived physiological indices - AMR and QFR - to concurrently evaluate microvascular and epicardial coronary function in a real-world primary PCI setting. AMR serves as an angiography-derived surrogate of coronary microvascular resistance and has been evaluated against invasive indices such as IMR (6, 9). In the present study, nicorandil treatment reduced AMR by more than 50%, indicating a substantial improvement in microvascular perfusion.

Unlike IMR, AMR allows immediate post-PCI assessment without pressure wires or pharmacological hyperemia, which is particularly advantageous in the emergent STEMI setting. Consistently, higher Vessel QFR values and lower *Δ*QFR further confirmed improved epicardial physiological flow and reduced translesional pressure gradients. Because QFR incorporates three-dimensional vessel reconstruction and flow dynamics, it provides a more comprehensive physiological assessment than conventional angiography alone (5, 7). Taken together, the parallel improvements in AMR and QFR highlight the dual beneficial effects of nicorandil on microvascular resistance and epicardial coronary conductance.Because AMR is derived from angiography-based flow modeling and is therefore not independent of QFR, we interpret concordant changes as internally consistent physiological signals rather than fully independent confirmations of treatment effect.

### Attenuation of myocardial injury and ischemia-reperfusion damage

Patients treated with intracoronary nicorandil showed numerically lower peak CK-MB and cTnI levels during hospitalization, suggesting attenuation of myocardial necrosis and ischemia-reperfusion injury. Although these differences did not reach statistical significance, the trend is biologically plausible and consistent with the observed improvements in microvascular perfusion reflected by AMR and QFR.

Several mechanisms may explain this cardioprotective effect. Nicorandil preserves microvascular integrity by reducing endothelial swelling, leukocyte adhesion, and capillary obstruction, thereby limiting microvascular obstruction (13). Activation of mitochondrial KATP channels stabilizes mitochondrial membrane potential and prevents opening of the mitochondrial permeability transition pore, a central mediator of reperfusion-related apoptosis and necrosis (14). Nicorandil also reduces intracellular calcium overload, limiting myocyte hypercontracture and cell death (15), while attenuation of oxidative stress and inflammatory responses may further restrict infarct expansion (16).

Although the present study cannot establish causality, the parallel improvement in angiography-derived microvascular indices and the trend toward lower myocardial enzyme release support a mechanistic link between microvascular protection and attenuation of myocardial injury. Although early improvement in left ventricular ejection fraction did not reach statistical significance at one week, these biochemical findings suggest the potential for more favorable long-term ventricular remodeling, as reported previously (17).

### Why early LVEF may not improve despite better microvascular indices

We acknowledge that this pilot study was underpowered to detect differences in clinical outcomes (MACE) or early LVEF. Improvements in angiography-derived microvascular indices (AMR/QFR) primarily indicate restoration of perfusion and alleviation of microvascular obstruction, which may reduce downstream injury and adverse remodeling over time (9, 12).However, early LVEF assessed at approximately one week may not immediately reflect microvascular recovery because (i) myocardial stunning after ischemia–reperfusion can cause transient, reversible contractile dysfunction with delayed functional recovery (12, 18), (ii) baseline infarct size and irreversible necrosis established before PPCI strongly influence early global systolic function (12, 19), and (iii) ventricular remodeling is a progressive process, and perfusion recovery is not sufficient by itself to guarantee early functional improvement (18, 20, 21).Therefore, longer follow-up with outcome-linked validation and imaging-based endpoints (e.g., CMR for infarct size and microvascular obstruction) is warranted to determine whether the observed physiological improvements translate into long-term functional and clinical benefit (12, 18, 22).

### QFR and AMR as practical tools for microcirculatory assessment in STEMI

Conventional physiological indices, including FFR and the IMR, require pressure wires, pharmacological hyperemia, and additional procedural time, which limits their routine use during emergent primary PCI. In contrast, QFR and AMR enable rapid, wire-free, and adenosine-free assessment of coronary physiology using standard angiographic data (6, 9).

Our findings support the feasibility and sensitivity of these angiography-derived indices for detecting treatment-related improvements in both epicardial and microvascular function. In addition to their diagnostic value, QFR and AMR may serve as quantitative markers of therapeutic response, which is increasingly important as microcirculation-targeted therapies continue to develop.

### Safety profile of intracoronary nicorandil

Despite concerns regarding hemodynamic instability with vasodilator therapy, low-dose intracoronary nicorandil did not increase the incidence of intraoperative hypotension in this study. In addition, in-hospital and 3-month major adverse cardiovascular events did not differ significantly between groups. These findings suggest no safety signal in this pilot cohort and are broadly consistent with prior reports indicating tolerability of nicorandil in acute myocardial infarction settings (23).

In contemporary practice, intracoronary or systemic vasodilators are commonly used to improve microvascular perfusion; however, vasodilatory therapies may provoke clinically relevant blood pressure reductions in susceptible patients. Nitroglycerin can lower blood pressure in a dose-dependent manner and may contribute to hemodynamic fluctuations (24).More broadly, selection of potent vasodilators in acute cardiovascular settings requires attention to hypotension risk and hemodynamic tolerance (25). Accordingly, peri-procedural hemodynamic stability is an important consideration when selecting adjunctive therapies during primary PCI.

Prior studies suggest that nicorandil may offer a hemodynamically tolerable option in selected settings. In patients with acute heart failure and low baseline systolic blood pressure, intravenous nicorandil did not increase hypotension risk while improving short-term outcomes (26). In the perioperative setting of primary PCI for STEMI, intravenous nicorandil reduced infarct size without increasing hypotension within 24 h (27). Consistent with these observations, in our cohort intracoronary nicorandil was not associated with an increased risk of peri-procedural hypotension or short-term adverse events, and in-hospital and 3-month MACEs did not differ significantly between groups.

With respect to efficacy, intracoronary nicorandil was associated with improvements in angiography-derived surrogate indices (AMR and QFR). However, these indices should be interpreted as surrogate physiological signals rather than definitive “microcirculatory function using angiography-derived surrogates” and our study was not powered to detect differences in clinical outcomes. Consistent with our multiplicity-controlled analyses, while key physiological indices remained significant after BH-FDR correction, clinical events and several secondary outcomes were not significant after correction, supporting cautious interpretation.Prior randomized and meta-analytic evidence suggests that nicorandil may reduce no-reflow/microcirculatory resistance and may improve remodeling or longer-term outcomes after STEMI (23, 28, 29), with additional supportive conference-level evidence from meta-analytic data (30). Larger, adequately powered multicenter studies with longer follow-up are required to confirm outcome-linked clinical benefit.

### Comparison with commonly used no-reflow vasodilators

Agents such as nicardipine and nitroprusside are widely used to treat no-reflow and primarily act as potent vasodilators to relieve microvascular spasm. Nicorandil has a distinct pharmacologic profile with dual actions (nitrate-like vasodilation and KATP channel opening), which may provide microvascular protection beyond vasodilation alone and potentially influence reperfusion injury pathways. Importantly, our study did not include an active comparator arm (e.g., nicardipine or nitroprusside); therefore, we cannot claim superiority. Rather, our findings suggest a feasible adjunctive option with a distinct mechanism, which merits future head-to-head comparative trials in contemporary practice.

### Limitations

Several limitations warrant consideration. First, this single-center study included a relatively small sample size, which may limit the generalizability of the findings. Second, the follow-up period was limited to three months and primarily captured short-term safety and early events after primary PCI, without enabling assessment of longer-term ventricular remodeling or late clinical outcomes. Longer follow-up (e.g., ≥ 12 months) and imaging-based endpoints (e.g., CMR for infarct size and microvascular obstruction) will be important in future multicenter studies; longer-term follow-up is ongoing in our cohort and will be reported in future work.

Third, we used a fixed intracoronary dose of nicorandil; alternative dosing regimens or repeated administration were not examined. Drug availability may differ across regions and institutions, and nicorandil is not routinely stocked in all catheterization laboratories; this pragmatic consideration may affect external applicability and supports the need for multicenter evaluation in diverse practice settings.Although nicorandil has been investigated in prior STEMI/AMI PCI studies, its availability varies across institutions, which may limit immediate generalizability (22, 31–34).

Fourth, AMR and QFR are angiography-derived indices that depend on image quality, acquisition technique, and dedicated software availability. Availability of dedicated software and standardized angiographic acquisition required for AMR/QFR analysis may vary across catheterization laboratories, which could limit immediate generalizability. Wider implementation may require standardization of acquisition protocols and, ideally, core-lab or multicenter validation. In addition, QFR/AMR analyses were performed locally and not adjudicated by an independent core laboratory, which may limit external reproducibility.Larger multicenter studies with standardized imaging protocols are needed to confirm and extend these results.

Finally, angiography-derived physiological indices are surrogate measures and do not by themselves establish clinical benefit. In addition, obtaining written consent in an emergency setting may preferentially include more stable patients and could introduce selection bias, potentially limiting generalizability.

### Clinical implications and future perspectives

From a clinical perspective, intracoronary nicorandil may represent a feasible and potentially beneficial adjunctive strategy to improve surrogate physiological measures of reperfusion during primary PCI in STEMI, while definitive clinical efficacy requires confirmation in adequately powered trials. This approach may be particularly useful in patients at high risk of microvascular dysfunction, such as those with prolonged ischemic time or a high thrombus burden. In addition to the therapeutic role of nicorandil, this study emphasizes the clinical utility of angiography-derived physiological indices for assessing microcirculatory function using angiography-derived surrogates in contemporary STEMI management.

Future investigations should include larger multicenter randomized trials, integration of advanced imaging modalities such as cardiac magnetic resonance to quantify microvascular obstruction and infarct size, and optimization of nicorandil dosing and delivery strategies. The integration of multimodal physiological assessment may ultimately enable more individualized reperfusion strategies and improve long-term outcomes.

## Conclusion

Intracoronary administration of 2 mg nicorandil during primary PCI in patients with first-episode STEMI was associated with lower angiography-derived microcirculatory resistance, improved QFR-based physiological indices, and a higher proportion of TIMI grade 3 flow, without an observed increase in intraoperative hypotension or short-term adverse cardiovascular events. Nicorandil also showed a consistent favorable trend toward reduced periprocedural myocardial injury. These findings reflect improvements in angiography-derived surrogate measures and should be interpreted as exploratory; definitive clinical efficacy requires confirmation in adequately powered, multicenter trials with longer follow-up and outcome-linked validation.

## Data Availability

The original contributions presented in the study are included in the article/Supplementary Material, further inquiries can be directed to the corresponding authors.
