# Supplementary material for: Intracoronary nicorandil improves coronary microcirculatory function after primary PCI in first-episode STEMI: an angiography-derived evaluation using AMR and QFR
Source: Front Cardiovasc Med. 2026 Apr 22;13:1786130. doi: 10.3389/fcvm.2026.1786130 (PMC13143652; doi:10.3389/fcvm.2026.1786130)
Supplement: Supplementary file 1 [file Table1.docx]

**Supplementary Table S1 . Firth penalized logistic regression for inadequate STR (≤70%)**

| Predictor | Primary exploratory model OR (95% CI), P | Extended exploratory model OR (95% CI), P | Sensitivity exploratory mode OR (95% CI), P | Further extended exploratory model OR (95% CI), P |
| --- | --- | --- | --- | --- |
| Nicorandil vs Control | 0.399 (0.107–1.345), 0.140 | 1.549 (0.282–10.688), 0.619 | 1.163 (0.189–8.250), 0.870 | 3.435 (0.240–67.970), 0.367 |
| Age (per 1 year) | 1.071 (1.018–1.138), 0.006 | 1.091 (1.012–1.206), 0.020 | 1.104 (1.019–1.234), 0.012 | 1.096 (1.014–1.222), 0.017 |
| TIMI 3 vs <3 (post-PCI) | — | 0.004 (0.00002–0.066), <0.001 | 0.005 (0.00003–0.074), <0.001 | 0.005 (0.00003–0.078), <0.001 |
| Pre-PCI TIMI 0 vs ≥1 | — | — | 0.299 (0.039–1.888), 0.198 | — |
| AMR (per 1 unit) | — | — | — | 2.001 (0.348–16.117), 0.449 |

Outcome:

Inadequate STR (≤70%; PR/NR) versus complete STR (>70%; CR).

Note:

Models were estimated using Firth penalized logistic regression due to sparse cells/separation in post-PCI TIMI categories under maximum-likelihood estimation. OR, odds ratio; CI, confidence interval; STR, ST-segment resolution; AMR, angiography-derived microcirculatory resistance; TIMI, Thrombolysis in Myocardial Infarction; PCI, percutaneous coronary intervention.
