# Supplementary figures and images for: Intracoronary nicorandil improves coronary microcirculatory function after primary PCI in first-episode STEMI: an angiography-derived evaluation using AMR and QFR
Source: Front Cardiovasc Med. 2026 Apr 22;13:1786130. doi: 10.3389/fcvm.2026.1786130 (PMC13143652; doi:10.3389/fcvm.2026.1786130)

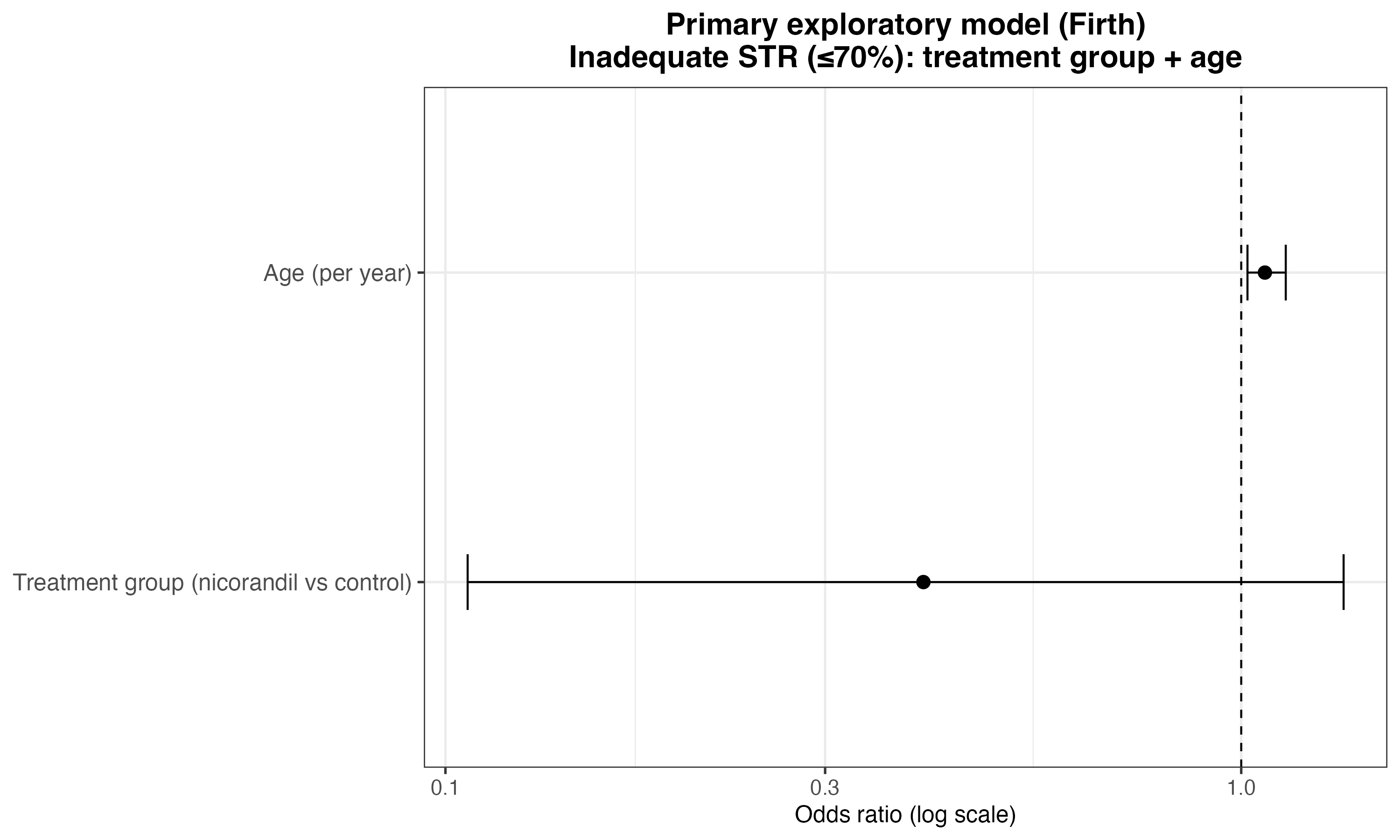

Supplement: Supplementary file 2 [file Image1.png]

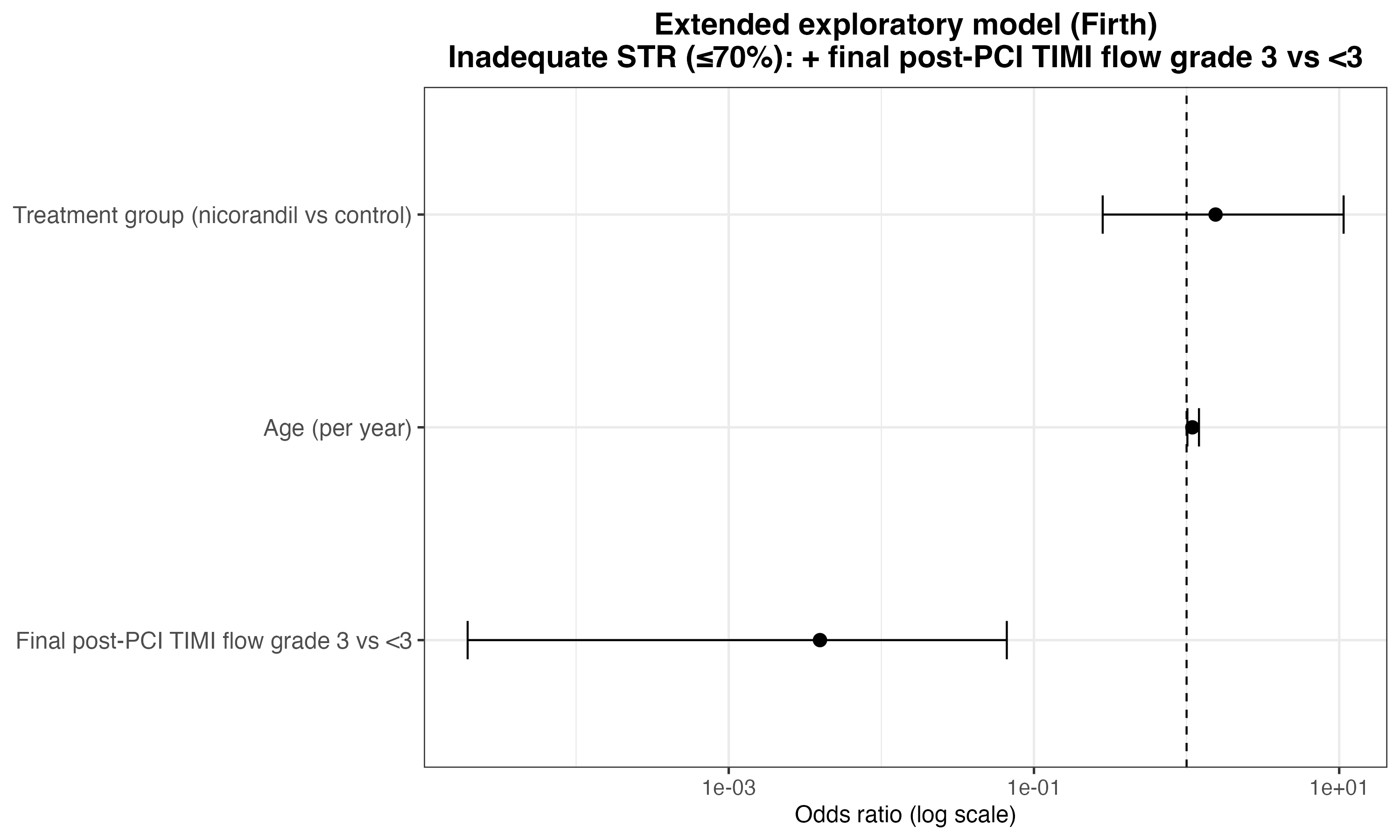

Supplement: Supplementary file 3 [file Image2.png]

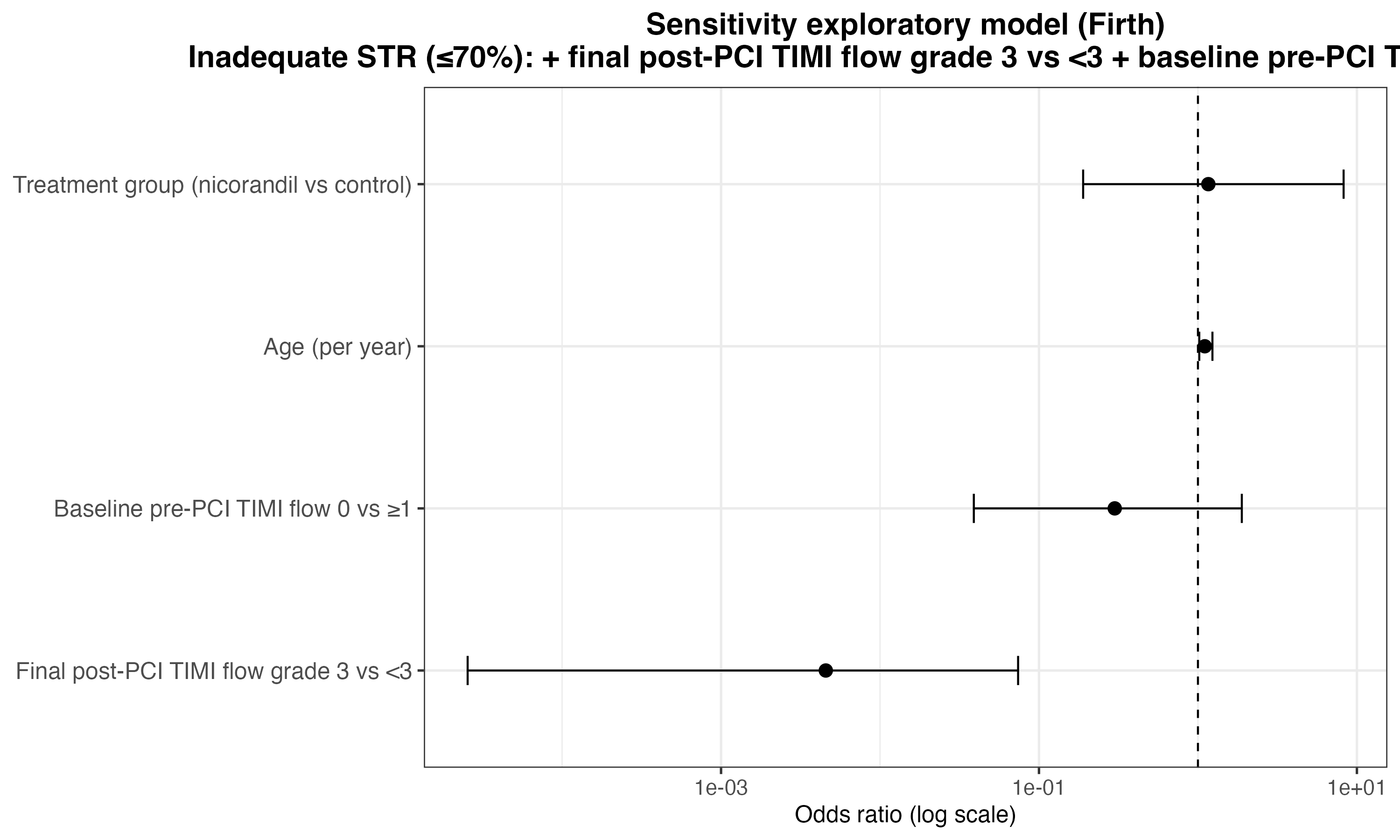

Supplement: Supplementary file 4 [file Image3.png]

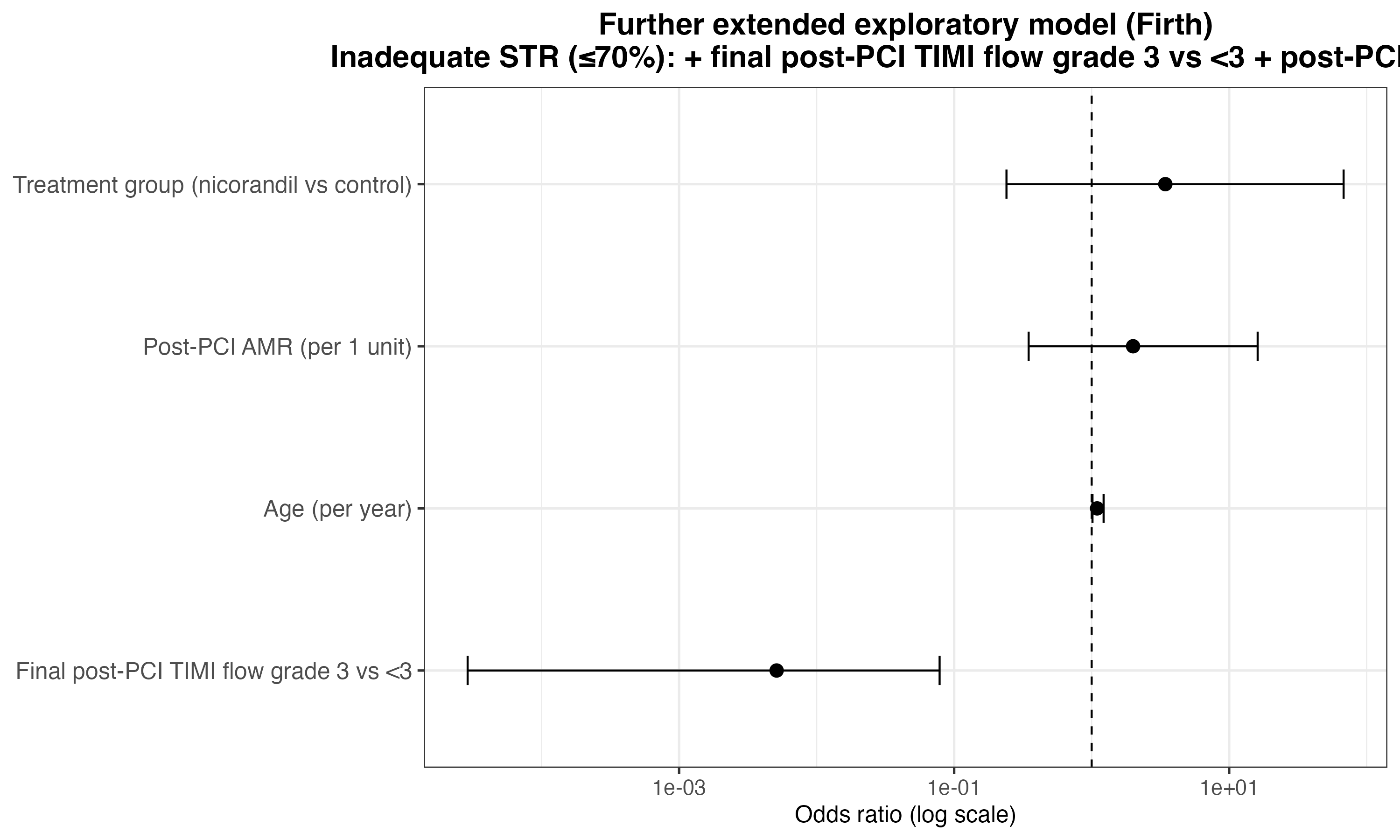

Supplement: Supplementary file 5 [file Image4.png]
